# Effect of diagnosis delay on pulmonary function in children with asthma

**DOI:** 10.1186/s13223-022-00731-w

**Published:** 2022-10-19

**Authors:** Xiaoling Wei, Min Xue, Jinyan Yan, Yuling Han, Yanqin Liu, Miao Liu, Jing Sun, Yun Zhang, Lu Cheng, Xiang Ma, Zhongtao Gai

**Affiliations:** 1 Department of Respiratory Diseases, Children’s Hospital Affiliated to Shandong University, Jinan Children’s Hospital, Jinan, 250022 Shandong China; 2 Jinan Institute of Pediatric Research, Children’s Hospital Affiliated to Shandong University, Jinan Children’s Hospital, Jinan, 250022 Shandong China; 3 Key Laboratory of Respiratory Disease for Children, Jinan Children’s Hospital, Jinan, 250022 Shandong China

**Keywords:** Asthma, Delayed diagnosis, Pulmonary function, Children

## Abstract

**Background:**

The effects of a delayed diagnosis of asthma on lung function in children have not been well investigated. Therefore, a retrospective cohort study was conducted in a children’s hospital to analyse the effect of delayed diagnosis time on lung function in children with asthma.

**Methods:**

We conducted a retrospective cohort study in Jinan Children's Hospital from January 1, 2010, to December 31, 2020. All children were divided into different groups according to the presence or absence of rhinitis, age at first onset (first coughing and wheezing attack) and delayed diagnosis duration (≤ 3 months, 3–12 months, 1–3 years, 3–5 years and > 5 years).

**Results:**

A total of 1,014 children with asthma were included in this study. The median (quartile) delay in asthma diagnosis among all participants was 11 (2, 26) months. The shortest delay in diagnosis time was on the same day of onset, and the longest delay in diagnosis time was 10 years. The median (quartile) duration of delayed diagnosis was 10 (2, 26) months in 307 asthmatic children without rhinitis and 11 (2, 26) months in 707 children with asthma and rhinitis (*P* < 0.05). The delayed diagnosis time was shorter among female children than among male children (*P* < 0.05), and the first %predicted forced volume capacity (FVC%pred) results for females were higher than those for males (*P* = 0.036). The children whose age at first asthma onset was ≤ 3 years had a longer delayed diagnosis duration than those whose age at first onset was > 3 years (*P* < 0.05). The FVC%pred and %predicted forced expiratory volume in 1 s (FEV1%pred) in the first and second pulmonary function tests were significantly lower in the five delayed diagnosis groups (all *P* < 0.05). After standardised treatment for 3–6 months, FVC%pred showed a significant difference in the third test among the 5 groups (*P* < 0.05), but the other pulmonary function indices showed no significant difference. Logistic regression analysis showed that longer delay and young age of onset were associated with lower lung function (*P* < 0.05), whereas sex, rhinitis and eczema had no significant effects (all *P* > 0.05) on FVC%pred and FEV1%pred.

**Conclusion:**

Although delayed asthma diagnosis can lead to lung function impairment in children with asthma, lung function can be improved quickly after standardised treatment. Therefore, early asthma diagnosis and standardised treatment are very important.

## Introduction

Asthma is a serious global health problem that affects all age groups; it is the most frequent chronic respiratory disease in children and the leading cause of school absences, emergency department visits and hospitalisations among children [1, 2]. Asthma is a heterogeneous disease characterised by airway inflammation. The standard definition is a history of respiratory symptoms, such as cough, wheezing, shortness of breath and chest tightness, that are recurrent at night and/or early in the morning [3]. National and international scholars have made great efforts to reduce the underdiagnosis of asthma. However, the correct diagnosis of asthma remains an urgent issue for improving the quality of life of children. Moreover, the lack of timely diagnoses causes an increase in morbidity and mortality [4]. A delay in asthma diagnosis in children results in the delay of appropriate treatment. Children may avoid exercise, miss school and be less productive, and their quality of sleep and overall quality of life are likely to be adversely affected by asthma. A long delay in diagnosis time is likely to result in patients with ongoing symptoms (potentially serious), exacerbations and long-term airway remodelling [1]. Indeed, diagnosis delay in children with asthma can seriously influence children’s physical and mental development, aggravate disease severity, reduce lung function, lower the quality of life and increase the cost and burden to patients’ health and health care systems [5]. Delays in the early diagnosis of asthma in children ≤ 5 years are associated with variable and nonspecific clinical symptoms, poor knowledge of primary health care providers, and difficulties in performing lung function and other asthma tests [6]. There are few studies on the impact of delayed asthma diagnosis on lung function and a lack of long-term follow-up studies on large cohorts, especially among children. Therefore, we designed this study to evaluate delayed asthma diagnoses among Chinese children and their impact on lung function. To our knowledge, this study is the largest diagnostic–prognostic study on the use of lung function to ascertain asthma cases in hospitals or the general population.

## Participants and methods

### Study design and setting

This was a retrospective cohort study conducted in the Department of Respiratory and Asthma Centre of Jinan Children's Hospital from January 1, 2010, to December 31, 2020.

### Ascertainment of asthma cases

The diagnosis of asthma was based on the guidelines for the diagnosis and treatment of bronchial asthma in children (2016), and improvement in asthma control was conducted in accordance with the Global Initiative for Asthma Prevention (GINA) and Treatment [3, 7]. The inclusion criteria for this study were children aged 5–16 years with confirmed asthma who had received long-term standardised asthma treatment at our hospital for at least 6 months and had completed 3 or more pulmonary function tests as prescribed by a respiratory specialist. Long-term standardised treatment of asthma is defined as a step-up or step-down treatment strategy based on control levels in accordance with the GINA guidelines for the use of ICS or a combination of other medications [7]. Children with congenital airway or vascular abnormalities, chronic airway or lung disease, unsuitability for pulmonary function testing, and failure to adhere to follow-up and treatment were excluded.

Clinician-diagnosed rhinitis was defined as the presence of one or more of the following symptoms: nasal congestion, rhinorrhoea, sneezing and/or posterior nasal drainage; allergic rhinitis was diagnosed when rhinitis symptoms were caused by an immunoglobulin E (IgE)-mediated process. All the patients in the present study were confirmed to have allergic rhinitis (i.e., atopic) by skin testing and/or serum-specific IgE measurements.

### Analysed variables

The analysed variables included demographic data, clinical data and laboratory measurements. Demographic data included age, sex and asthma incidence date. Clinical data included the presence or absence of rhinitis symptoms and itching eczema symptoms (current or past). Laboratory measurements included FVC%pred, FEV1%pred, FEV1/FVC% ratio, peak expiratory flow in percent predicted values (PEF%pred), peak expiratory flow in percent predicted values of maximal flow at 50% of VC (FEF50%pred) and peak expiratory flow in percent predicted values of maximal flow at 75% of VC (FEF75%pred).

The asthma incidence date was the earliest presence of symptoms that met the predefined criteria for asthma regardless of whether clinicians diagnosed the patient with asthma. The final diagnosis of asthma was made by respiratory specialists. All available retrospective data from complete medical records were analysed.

Pulmonary function testing was performed with a MasterScreen PFT System (Jaeger Company, Germany). Respiratory flow and volume were measured with a pressure screen-type pneumotachograph that was calibrated daily. All measurements were performed by trained investigators. The timing of each pulmonary function examination performed by the physicians was documented and analysed. The first pulmonary function examination was performed when asthma was diagnosed, the second examination was selected as the best lung function within 1–3 months, and the third examination was performed within 3–6 months after standardised treatment.

Delayed asthma diagnosis time was defined as the period between the date of the onset of symptoms (cough, wheezing, shortness of breath and chest tightness) and the date of diagnosis. The duration of undiagnosed asthma is age dependent; therefore, we divided our study population into five groups according to delayed asthma diagnosis time (≤ 3 months, 3–12 months, 1–3 years, 3–5 years and > 5 years) to enable each participant to contribute equally to the analysis.

In clinical work, we found that asthma/asthma plus rhinitis and onset of symptoms < 3 years versus > 3 years were important factors associated with lung function. All children with asthma included in this study were divided into two groups, namely, the asthma without rhinitis group and the asthma plus rhinitis group, according to whether they had rhinitis. At the same time, all children were also divided into two groups according to age at first onset of asthma symptoms, namely, the ≤ 3 years group and the > 3 years group.

### Data analysis

All data and additional information were obtained from the medical documentation of the children in our hospital. The delayed diagnosis time was consistent with a skewed distribution and described by the median (quartile) [M (P25, P75)]. The correlations of age, sex, clinical data and laboratory measurements with delayed versus nondelayed asthma diagnosis were assessed by logistic regression. Logistic regression was used to assess the relationship between a dependent variable (delayed asthma diagnosis) and an independent variable. Then, all independent variables were included in one model to identify the best predictor of the dependent variable (multivariate analysis). All statistical analyses were performed using SPSS 21.0.

## Results

### Demographic data

A total of 1014 patients with asthma were included in this study, including 673 males and 341 females aged 5–16 years, and the mean ± SD was 9.12 ± 2.33. The asthma without rhinitis group had 307 patients, including 187 males and 120 females aged 9.04 ± 2.35 years. The asthma with rhinitis group had 707 patients, including 486 males and 221 females aged 9.15 ± 2.32 years. A total of 249 patients (169 males and 80 females aged 8.52 ± 2.02 years) had their asthma onset at ≤ 3 years of age, and 765 patients (504 males and 261 females aged 9.31 ± 2.39 years) had their asthma onset at > 3 years of age.

The distribution of the study population and the basic information of children with different delayed asthma diagnosis times (in months) are shown in Table 1.

**Table 1:** Distribution of the study population in all variables

Duration of delayed asthma diagnosis	*N*	Male (*n*/%)	Asthma without rhinitis (*n*/%)	Asthma with rhinitis (*n*/%)	Onset ≤ 3 years (*n*/%)	Onset > 3 years (*n*/%)
≤ 3 months	315	205 (65.1)	102 (53.7)	213 (67.6)	35 (11.1)	280 (88.9)
3–12 months	261	159 (60.9)	70 (64.2)	191 (73.2)	40 (15.3)	221 (84.7)
1–3 years	260	177 (68.1)	78 (46.9)	182 (70.0)	68 (26.2)	192 (73.9)
3–5 years	115	80 (69.6)	40 (43.8)	75 (65.2)	62 (53.9)	53 (46.1)
> 5 years	63	52 (82.5)	17 (21.2)	46 (73.0)	44 (69.8)	19 (30.2)
Total	1014	673 (66.4)	307 (50.7)	707 (69.7)	249 (24.6)	765 (75.4)

### Influence of rhinitis on lung function in the first test

We analysed whether rhinitis was associated with the lung function of children with a delayed asthma diagnosis. The median (quartile) missed diagnosis time among 707 children with asthma and rhinitis was 11 (2, 26) months, which was significantly higher than that among the 307 children with asthma (10 [2, 26] months; Z =  − 26.21, *P* < 0.05). The asthma without rhinitis group and asthma with rhinitis group showed no significant differences in FVC%pred (101.83 ± 13.58 *vs.* 101.4 ± 14.03, *t* = 0.408, *P* = 0.68) or FEV1%pred (98.71 ± 16.00 *vs.* 98.07 ± 16.19, *t* = 0.584, *P* = 0.56).

### Influence of sex on lung function in the first test

The median (quartile) delay in asthma diagnosis time among the 341 female children was 9 (2.0, 24.0) months, which was shorter than that among the 673 male children (12 [3.0, 31.5] months; Z =  − 26.94, *P* < 0.05). The FVC%pred of the male children was lower than that of the female children (100.91 ± 13.72 *vs.* 102.85 ± 14.15, *t* =  − 2.11, *P* = 0.036), whereas FEV1%pred (97.84 ± 15.87 *vs.* 99.11 ± 16.62, *t* =  − 1.19, *P* = 0.24) showed no significant difference between males and females.

### Influence of age at asthma onset on lung function in children

The median (quartile) duration of undiagnosed asthma in the onset ≤ 3 years of age group was 31 (11.5, 54) months, which was longer than that of the onset > 3 years of age group (7 [2, 19] months; *Z* =  − 25.51, *P* < 0.05). No significant differences in the first FVC%pred and FEV1%pred results were found between the two groups (*t* = 0.30, *P* = 0.76; *t* = 0.51, *P* = 0.61).

### Influence of delayed diagnosis time on lung function in children with asthma

The median (quartile) duration of delayed diagnosis for all subjects was 11 (2, 26) months, the shortest diagnosis delay was on the day of onset, and the longest diagnosis delay was 10 years. The differences in the first FVC%pred (*F* = 4.91, *P* = 0.027) and FEV1%pred results (*F* = 5.02, *P* = 0.025) of the five diagnosis delay groups were statistically significant, but no significant differences in FEV1/FVC% (*F* = 1.11, *P* = 0.29), PEF%pred (*F* = 1.35, *P* = 0.24), FEF50%pred (*F* = 0.80, *P* = 0.37) or FEF75%pred (*F* = 2.62, *P* = 0.11) were observed among the five groups. After 1–3 months of standardised anti-asthma treatment, the second comparison of lung function in the five groups also showed significant differences in FVC%pred (*F* = 40.99, *P* = 0.001) and FEV1%pred (*F* = 9.68, *P* = 0.002) but not in FEV1/FVC% (*F* = 1.18, *P* = 0.28), PEF%pred (*F* = 0.97, *P* = 0.33), FEF50%pred (*F* = 2.29, *P* = 0.13) or FEF75%pred (*F* = 2.99, *P* = 0.08). The third comparison of lung function indices in the 5 groups showed statistically significant differences in FVC%pred (*F* = 4.51, *P* = 0.034) but not in the other lung function indices. All results are shown in Table 2.

**Table 2:** Three comparisons of pulmonary function in children with different delayed asthma diagnosis time

Duration of delayed asthma diagnosis	FVC% pred	FEV1% pred	FEV1/FVC%	PEF% pred	FEF50% pred	FEF75% pred
First comparison						
≤ 3 months	102.6 ± 13.2	99.5 ± 14.9	99.6 ± 7.7	91.6 ± 16.2	76.0 ± 20.9	88.0 ± 19.6
3–12 months	101.6 ± 14.4	98.1 ± 16.9	96.1 ± 7.9	90.1 ± 18.1	74.8 ± 23.1	86.2 ± 21.8
1–3 years	101.6 ± 13.7	98.5 ± 16.7	96.2 ± 8.9	90.8 ± 17.5	75.7 ± 24.2	87.1 ± 22.1
3–5 years	100.3 ± 14.8	96.6 ± 16.8	95.6 ± 9.5	89.5 ± 17.9	73.5 ± 23.8	84.8 ± 23.1
> 5 years	98.5 ± 13.8	94.8 ± 14.8	95.7 ± 8.1	89.2 ± 14.9	73.9 ± 21.5	83.6 ± 19.5
*F*	4.9	5.0	1.1	1.4	0.8	2.6
*P*-value	0.03	0.03	0.3	0.2	0.4	0.1
Second comparison						
≤ 3 months	104.3 ± 12.0	102.4 ± 2.8	97.9 ± 6.4	95.7 ± 15.1	82.4 ± 20.1	93.4 ± 17.9
3–12 months	103.1 ± 12.5	101.4 ± 14.0	98.2 ± 6.9	95.0 ± 15.7	82.5 ± 21.5	92.9 ± 20.4
1–3 years	103.0 ± 12.4	100.9 ± 14.8	97.5 ± 8.5	95.6 ± 16.4	80.9 ± 23.6	91.6 ± 21.3
3–5 years	102.2 ± 10.8	100.2 ± 12.3	97.7 ± 7.4	94.6 ± 14.3	80.1 ± 19.7	92.3 ± 18.6
> 5 years	98.0 ± 13.6	96.0 ± 15.5	96.9 ± 7.5	93.2 ± 4.4	79.1 ± 21.8	88.2 ± 21.3
*F*	11.0	9.7	1.2	0.97	2.3	3.0
*P*-value	0.001	0.002	0.28	0.33	0.1	0.08
Third comparison						
≤ 3 months	103.0 ± 11.7	101.5 ± 12.7	98.4 ± 6.9	97.0 ± 15.2	83.2 ± 20.7	93.9 ± 19.2
3–12 months	103.6 ± 12.1	101.5 ± 12.8	97.9 ± 6.4	96.8 ± 14.2	82.6 ± 20.5	93.7 ± 18.4
1–3 years	102.1 ± 12.2	101.2 ± 13.3	98.5 ± 7.7	96.4 ± 15.9	83.6 ± 22.1	94.1 ± 20.7
3–5 years	102.6 ± 13.0	101.2 ± 14.1	98.2 ± 7.1	97.7 ± 5.6	82.5 ± 18.9	95.7 ± 20.1
> 5 years	99.0 ± 10.6	97.0 ± 10.8	97.5 ± 6.4	95.2 ± 12.6	80.4 ± 18.4	89.9 ± 19.3
*F*	4.5	3.0	0.5	0.4	0.9	1.0
*P*-value	0.03	0.08	0.5	0.5	0.3	0.3

### Risk factors for reduced pulmonary function

We separately analysed the risk factors for reduced lung function in each age group. The correlations of age at onset, sex, delayed diagnosis time, rhinitis and itching eczema were included as independent variables in the multivariate model for logistic regression analysis. In all cases the lung function values FVC%pred and FEV1%pred were significant lower in those with greater diagnosis lag. Logistic regression analysis showed that different ages at onset and delayed diagnosis times showed statistically significant differences in FVC%pred and FEV1%pred (*P* < 0.05) but not in other lung function indices. All variables are shown in Tables 3 and 4.

**Table 3:** Risk factors that affect FVC%

Risk factors	Regression coefficients (95% CI)	Standard error	*t*	*P*
Constants	103.4 (99.6–107.3)	2.0	52.7	< 0.001
Age of onset (month)	− 0.04 (− 0.7–0.1)	0.02	− 2.7	0.006
Delayed diagnosis time (month)	− 0.07 (− 0.1–0.02)	0.02	− 2.9	0.003
Gender	1.7 (− 0.1–3.5)	0.9	1.8	0.07
Rhinitis	− 0.08 (− 2.0–1.8)	1.0	− 0.08	0.9
Itching eczema	− 0.8 (− 2.6–1.0)	0.9	− 0.9	0.4

**Table 4:** Risk factors that could affect FEV1%

Risk factors	Regression coefficients (95% CI)	Standard error	*t*	*P*
Constants	103.6 (99.2–108.1)	2.3	45.6	< 0.001
Age of onset (month)	− 0.07 (− 0.1–0.04)	0.02	− 4.0	< 0.001
Delayed diagnosis time (month)	− 0.09 (− 0.2–0.04)	0.03	− 3.7	< 0.001
Gender	0.9 (− 1.2–3.0)	1.1	0.8	0.4
Rhinitis	− 0.4 (− 2.6–1.8)	1.2	− 0.4	0.7
Itching eczema	− 0.6 (− 2.7–1.5)	1.1	− 0.5	0.67

## Discussion

Lung function examination, as an important part of the assessment of respiratory diseases, especially in asthmatic diseases, has great importance in the diagnosis, differential diagnosis, treatment and prognostic evaluation of the future risk of asthma. In the course of asthma diagnosis and treatment, lung function should be measured at the beginning of treatment, after 3–6 months of treatment (to identify the patient's personal best lung function values) and periodically thereafter for ongoing risk assessment [3, 8]. Regular lung function monitoring can provide a more comprehensive analysis of the severity and control status of asthma, which can provide an objective basis for precise treatment. Asthma diagnosis in young children is difficult to objectively establish, and asthma is often misdiagnosed as upper respiratory tract infection, bronchitis, asthmatic bronchitis, pneumonia or other diseases at an early stage. For these diseases, anti-infection treatment is carried out in accordance with infectious diseases, and the treatment ends when the symptoms are relieved. Because of the repeated diagnosis of respiratory infections, these children may be at risk for repeated prescriptions for antibiotics and chest X-rays. However, the effcet caused by repeated radiographs or treatment with antibacterial drugs is difficult to estimate [9]. The definition and diagnosis of asthma may be the subject of controversy in the case of individuals in their first years of life because of the difficulty of performing objective pulmonary function tests or the tendency of symptoms to subside during the course of childhood. This factor has led to the current situation wherein many children with asthma have not undergone spirometry testing, and the onset of asthma is frequently established by patient recall in epidemiological studies [10]. In the present study, we analysed the duration of delayed asthma diagnosis for 1014 patients aged 5–16 years and found that the longest asthma diagnosis delay was up to 10 years. The age at onset and delayed diagnosis time were associated with more impairment in lung function. This finding suggests that a great gap in asthma diagnosis still exists despite the Chinese diagnostic criteria being followed, especially in primary hospitals, and even though great progress in the correct diagnosis and treatment of childhood asthma has been made. Early detection, patient education and optimal treatments are currently the main strategies for asthma management.

Allergic rhinitis (AR) has a strong association with asthma, which has led to the concepts of united airway disease and combined allergic rhinitis and asthma syndrome, or ‘one airway-one disease’. Researchers have advocated that these two conditions are different aspects of the same disease [11, 12]. Increasing evidence has shown that AR precedes the onset of asthmatic symptoms, and uncontrolled moderate to severe allergic rhinitis can affect asthma control considerably. The results of our study revealed that 707 of the 1014 cases (69.72%) were complicated with rhinitis. In a study by Broek and Bousquet on AR and its impact on asthma guidelines (2016 Revision), they noted that nasal symptoms are present in 6%–85% of patients with asthma. Our study showed that the delayed asthma diagnosis time in the asthma without rhinitis group was shorter than that in the asthma with rhinitis group. The presence of AR seems to reduce the diagnostic rate of asthma, probably because some of the treatments for AR management may mask the developing symptoms of asthma, which will reduce the diagnostic accuracy. Additionally, early asthma symptoms could be mistaken as rhinitis symptoms and may not be diagnosed in a timely manner. Therefore, strengthening the cooperation between the otolaryngology department and respiratory department or allergic department in clinical practice is necessary to improve the diagnostic level of asthma and rhinitis. Comfortingly, although the presence of asthma with rhinitis delayed the diagnosis time of asthma for children, no statistically significant difference in FVC%pred or FEV1%pred was found between the two groups. This result suggests that rhinitis is the most common disease that coexists with asthma, but it is not an independent risk factor for the reduction in lung function in children with asthma. A cross-sectional analytical study reported that lung function abnormalities are more prevalent among patients with moderate to severe persistent rhinitis and are associated with the frequency and severity of rhinitis [13]. Kessel also found that children with AR have impaired lung function, especially decreased FEF25%pred and FEF75%pred. Children with impaired lung function have a high prevalence of positive bronchodilation test results, which are considered important for asthma diagnosis. This finding suggests the presence of a continuum in children with AR from normal lung function towards asthma development [14]. AR is regarded as a risk factor for asthma development, especially in the presence of hyperresponsiveness. This occurrence may be explained by the presence of persistent nasal inflammation, which may be associated with the involvement of the lower airways with mucosal infiltration and lung function impairment [15].

Asthma is recognised as one of the most costly respiratory disorders that cause substantial disability, impaired quality of life and deaths among children [16]. Pulmonary function impairment associated with asthma usually begins at preschool age [17]. Recurrent wheezing in preschool children, especially in those under 6 years of age, is a very common issue; 50% of all children had at least one wheezing episode in the first 6 years of life [18]. Preschool wheezing is a heterogeneous condition, and different phenotypes have been described with considerable overlap in symptoms, such as infants and toddlers with atypical wheezing and multiple phenotypes, so identifying their atypical wheezing performance to characterise and manage these patients can be a great challenge. Moreover, lung function tests are difficult to implement within this age group. Thus, missed diagnoses and misdiagnoses are prone to occur and hinder opportunities for timely control [18, 19]. In the present study, we found that delayed asthma diagnoses lead to decreased lung function, which suggests that early assessment of asthma and active control of asthma in patients with recurrent wheezing are needed. We gathered cases with delayed asthma diagnoses in each age group; moreover, differences in FVC%pred and FEV1%pred at the first diagnosis of asthma were statistically significant among the five groups. After standardised treatment, significant differences in FVC%pred and FEV1%pred were still found in the second comparison of the pulmonary function indices of the five groups. In the third comparison of the pulmonary function indices, only FVC%pred had significant differences among the five groups. The results suggest that delayed asthma diagnosis has a certain impact on the lung function of children with asthma, but standard treatment could improve the impact of this delayed diagnosis.

Clinical practice has proven that standardised asthma treatment plays an important role in maintaining a normal level of lung function and improving the quality of life of children with asthma. In our previous study, wherein we conducted a 3-year follow-up of 198 children with asthma, we found that after standardised treatment, the lung function indices PEF%pred, FEF50%pred and FEF75%pred were improved significantly [20]. Asthma management programs aim to improve the health status of children with chronic disease and reduce associated costs. Improving the management of children with asthma to achieve a good level of lung function has become an important means to compensate for the reduced lung function caused by missed early diagnosis time. Self-management involves educating and enabling children to achieve good control of their asthma symptoms to reduce the risk of future exacerbations [21].

Asthma has a serious influence on children's physical and mental development, and the lack of a timely diagnosis and active standard treatment would aggravate the disease; however, an adequate prediction of asthma in these children is difficult and cannot be reliably assessed with conventional clinical tools. It is especially important to identify the high-risk factors for childhood asthma and establish a prediction model for childhood asthma. Our study revealed that the development of prophylactic strategies requires a good command of indications for urgent and long-term standardised treatment, and primary medical and health institutions should be included in strategies for the prevention and treatment of asthma.

In summary, the present study found that age at onset and delayed diagnosis time had a significant influence on FVC%pred and FEV1%pred, and a longer delay in diagnosis was associated with greater lung function impairment. Clinicians need to fully grasp the risk factors and typical clinical symptoms of asthma, including recurrent wheezing, coughing, shortness of breath and chest tightness, as well as various common causative factors. Furthermore, the cause and effect can be difficult to disentangle because although the presence of asthma can conceivably hinder lung growth, other processes impairing lung growth can also cause similar, nonspecific symptoms to those traditionally associated with asthma (dyspnoea, cough and wheezing). In the absence of an objective diagnostic measurement, such a child is likely to be diagnosed with and treated for asthma. Clinicians need to improve their ability to comprehensively analyse the illness; find further information about relevant medical history, especially family history, personal history and history of allergies; and combine specific and regular examinations in the diagnosis of asthma to ensure the clinical control and normal lung function of children with asthma. Importantly, developing diagnostic criteria for asthma in the absence of adequate lung function is imperative.

We recognise the limitations of our study. Our data were retrospective and cross-sectional. Thus, we have no measure of inhaler adherence between the two groups of patients, which might perhaps account for differences in control.

## Conclusions

To our knowledge, this study is the largest diagnostic–prognostic study on the use of lung function for asthma detection in the hospital or general population. We showed that delayed asthma diagnosis reduced lung function. Standard treatment can improve the influence of delayed diagnosis, and clinicians need to understand the diagnostic criteria, timely diagnosis and active standard treatment of asthma to promote physical and mental health and reduce disability among children with asthma.

## Data Availability

All data generated or analysed during this study are included in this article.

## References

[1] Kavanagh J, Jackson DJ, Kent BD (2019). Over- and under-diagnosis in asthma. Breathe (Sheff).

[2] Papi A, Brightling C, Pedersen SE, Reddel HK (2018). Asthma. Lancet.

[3] Global Initiative for Asthma. Global strategy for asthma management and prevention, 2020. www.ginaasthma.org.

[4] Côté A, Godbout K, Boulet LP (2020). The management of severe asthma in 2020. Biochem Pharmacol.

[5] Majak P, Bąk-Walczak E, Stelmach I, Jerzyńska J, Krakowiak J, Stelmach W (2011). An increasing trend of the delay in asthma diagnosis after the discontinuation of a population-based intervention. J Asthma.

[6] Brzozowska A, Majak P, Grzelewski T, Stelmach W, Kaczmarek J, Stelmach P (2009). Measurement of specific airway resistance decreased the risk of delay in asthma diagnosis in children. Allergy Asthma Proc.

[7] Yixiao B, Aihuan CH, Zhou F, Chongchang L, Chuanhe L (2016). Guidelines for the diagnosis and prevention of bronchial asthma in children (2016 edition). Chin J Pdeiatrics..

[8] Moral L, Vizmanos G, Torres-Borrego J, Praena-Crespo M, Tortajada-Girbés M, Pellegrini FJ (2019). Asthma diagnosis in infants and preschool children: a systematic review of clinical guidelines. Allergol Immunopathol (Madr).

[9] Lynch BA, Fenta Y, Jacobson RM, Li X, Juhn YJ (2012). Impact of delay in asthma diagnosis on chest X-ray and antibiotic utilization by clinicians. J Asthma.

[10] Agusti A, Faner R (2019). Lung function trajectories in health and disease. Lancet Respir Med.

[11] Giavina-Bianchi P, Aun MV, Takejima P, Kalil J, Agondi RC (2016). United airway disease: current perspectives. J Asthma Allergy.

[12] Paiva Ferreira LKD, Paiva Ferreira LAM, Monteiro TM, Bezerra GC, Bernardo LR, Piuvezam MR (2019). Combined allergic rhinitis and asthma syndrome (CARAS). Int Immunopharmacol.

[13] Saranz RJ, Agresta MF, Lozano NA, Alegre G, Sasia LV, Ianiero L (2019). Relationship between rhinitis severity and lung function in children and adolescents without asthma. Rev Fac Cien Med Univ Nac Cordoba.

[14] Kessel A (2014). The impact of intranasal corticosteroids on lung function in children with allergic rhinitis. Pediatr Pulmonol.

[15] Lambrecht BN, Hammad H (2013). Asthma: the importance of dysregulated barrier immunity. Eur J Immunol.

[16] Asher I, Pearce N (2014). Global burden of asthma among children. Int J Tuberc Lung Dis.

[17] Ducharme FM, Tse SM, Chauhan B (2014). Diagnosis, management, and prognosis of preschool wheeze. Lancet.

[18] Padem N, Glick RR (2019). The infant and toddler with wheezing. Allergy Asthma Proc.

[19] Ducharme FM, Dell SD, Radhakrishnan D, Grad RM, Watson WT, Yang CL (2015). Diagnosis and management of asthma in preschoolers: a Canadian Thoracic Society and Canadian Paediatric Society position paper. Can Respir J.

[20] Xiang M, Chun Y, Yun Zh, Jing S, Yanqin L (2020). A follow-up study of children with asthma treated with standardized theryapy. China Clinical Practical Medicine.

[21] Harris K, Kneale D, Lasserson T, McDonald VM, Grigg J, Thomas J (2019). School-based asthma self-management interventions for children and adolescents with asthma. Paediatr Respir Rev.

